# Symptoms and Diagnoses Prior to Suicide in Children and Young Adults—A Swedish Medical Record Review

**DOI:** 10.3390/ijerph23010105

**Published:** 2026-01-13

**Authors:** Anna-Lena Hansson, Per Johnsson, Sophia Eberhard, Erik Bergqvist, Elin Fröding Saric, Linda Karlsson, Sara Lindström, Margda Waern, Åsa Westrin

**Affiliations:** 1 Department of Child and Adolescent Psychiatry, Skåne University Hospital, SE-22185 Lund, Sweden; 2 Department of Psychology, Lund University, SE-22362 Lund, Sweden; 3 Department of Clinical Sciences, Child and Adolescent Psychiatry, Lund University, SE-22100 Varberg, Sweden; 4 Psychiatric Clinic, Hallands Sjukhus Varberg, Region Halland, SE-43281 Varberg, Sweden; 5 Department of Clinical Sciences, Psychiatry, Lund University, SE-22184 Lund, Sweden; 6 School of Health and Welfare, Jönköpings University, Region Jönköpings County, SE-55185 Jonkoping, Sweden; 7 National Centre for Suicide Research and Prevention (NASP), Department of Learning, Informatics, Management and Ethics (LIME), Karolinska Institutet, SE-17177 Stockholm, Sweden; 8 National Centre for Suicide Research and Prevention (NASP), Centre for Health Economics, Informatics and Health Services Research (CHIS), Stockholm Health Care Services, SE-17177 Stockholm, Sweden; 9 Department of Psychiatry, Lund, Skåne University Hospital, SE-22184 Lund, Sweden; 10 Department of Psychiatry and Neurochemistry, Institute of Neuroscience and Physiology, University of Gothenburg, SE-41345 Gothenburg, Sweden; 11 Department of Psychotic Disorders, Sahlgrenska University Hospital, Region Västra Götaland, SE-41345 Gothenburg, Sweden

**Keywords:** suicide, symptoms, diagnoses, children, adolescents, young adults, psychology, clinical implications, public health, Sweden

## Abstract

**Highlights:**

**Public health relevance—How does this work relate to a public health issue?**

- Preventing suicide in children and young adults is an important global public health issue. This work is relevant due to its focus on understanding youth suicidality to prevent suicide and strengthen well-being in the younger generations.
- This study investigated symptoms and all DSM diagnoses among a national cohort of children and young adults that died by suicide in 2015.

**Public health significance—Why is this work of significance to public health?**

- This work offers an understanding of the proportions of symptoms and diagnoses in children and young adults prior to suicide in youth; relatively few had a history of symptoms and diagnoses.
- This work is of significance to public health due to its discussion of the complex interplay between the inner and outer world of the individual, including the physical, emotional, and social life worlds.

**Public health implications—What are the key implications or messages for practitioners, policy makers, and/or researchers in public health?**

- The key implication for practitioners and policy makers in public health is the recommendation to consider the highlighted psychological aspects of mental health and well-being in building psychological resilience to suicide as well as improving challenging life situations to prevent suicide.
- The key implication for researchers in public health is the need to continue to incorporate various theoretical perspectives while further exploring the plurality of youth suicidality.

**Abstract:**

Suicide in children and young adults is a leading cause of premature mortality, and there is a need to develop a more profound understanding of the factors that contribute to these deaths. This study is part of the nationwide Retrospective Investigation of Health Care Utilization in Individuals who died by Suicide in Sweden 2015, conducted at Lund University, Sweden. The aim was to examine symptoms and diagnoses in children and young adults who died by suicide, as documented in their medical records at their last visits for primary care, somatic specialist care, or psychiatric care 24 months prior to suicide, and to apply contemporary psychological research in youth suicidality to the findings to formulate clinical implications. The proportions of symptoms and diagnoses in children (0–17 years), young adults (18–24 years), males, and females are described. The main symptoms noted in the cohort were depressive symptoms (28%), anxiety symptoms (26%), and pain (25%). The diagnoses predominately covered mental and behavioural disorders, and the most frequent of the mental and behavioural diagnoses were neurotic, stress-related, and somatoform disorders (32%) and mood (affective) disorders (29%). The diagnoses and symptoms were not sufficient to uncover suicidality in children and young adults. The clinical implications for alternative assessments and preventive interventions are discussed.

## 1. Introduction

Suicide is a leading cause of mortality among youth aged 10–24 years globally [1]. In current health care systems, including psychiatric services, symptoms and diagnoses are a focus, both in the assessment and the treatment of suicidality. Thompson et al. [2] found that most youth who died by suicide had no history of recognized suicidal thoughts or behaviour. Previous research has pointed out the need to improve assessments and interventions to prevent suicide. Liang et al. [3] argued against a one-size-fits-all intervention, instead suggesting that the interventions should meet the individual characteristics behind the suicidal ideation. It is recommended to include youth with a lived experience in the forming of interventions to overcome the obstacles of stigma [4], expanding the possibilities of preventing suicide. Focusing on individuals’ psychiatric disorders is probably too narrow of an approach to preventing suicide [5]. Several studies have highlighted that suicidality in youth often involves complex patterns of comorbidities and psychosocial vulnerabilities. Thompson et al. [2] found an elevated suicide risk among youth with an internalizing psychopathology in combination with externalizing problems, substance misuse, and medical disorders. Similarly, Lannoy et al. [6] found that the risk of suicidal behaviour increased with alcohol use.

In suicidality, both internal and external challenging experiences are important, as well as their intensity and duration. Several authors have underscored the accumulation of these factors in combination with a lack of solutions to these troubles. Unmet interpersonal needs and psychache may partly explain why childhood trauma increases the risk of suicidal ideation [7]. Childhood trauma also relates to feelings of shame, emptiness, and rejection [7,8]. Commisso et al. [9] compared externalizing problems (rule-breaking, opposition, aggression), internalizing problems (depression, social withdrawal, anxiety), and the comorbidity of these problems in childhood and found that externalizing or both internalizing and externalizing problems gave a two- to three-times-higher risk of attempting suicide by 22 years than low/no problems in childhood. Shneidman [10] summarized the experiences leading to psychache as thwarted love, fractured control, an assaulted self-image, excessive anger, overwhelming shame, ruptured key relations, and attendant grief leading to frustrated needs and the inner pain of that turmoil. Pompili et al. [11] argued that the accumulation of stressors is the central feature prior to a suicide attempt. Therefore, they recommend suicide prevention early in the course of the development of suicide risk [11]. This psychological knowledge can be used to form assessments and preventive interventions.

External aspects that are of relevance in suicidality yield ongoing and prior negative life experiences. It is important to give attention to a stressful life, family conflict, and divorce, especially interpersonal problems, legal issues, and substance abuse [12]. In a study by Janiri et al. [13], children with self-reported family conflict were 30–75 percent more likely to experience suicidality. Aggressive marital conflict practices can affect the suicidality risk in children [14]. Panisch and Duprey [15] found that a mother’s negative experiences may negatively impact their parental capacity to recognize and respond to danger to their child [15]. Mothers with a higher degree of betrayal trauma were more likely to have maltreated children and children with dissociative symptoms, leading to a higher risk of suicidal ideation for their children at the age of 16 years [15]. Child maltreatment and family dysfunction have a significant effect on adult suicidal behaviour [16].

Good psychological development is preventive of suicide by forming well-being and resilience. If not formed in childhood through supporting parenting and family connectedness, mental well-being and resilience can be built or rebuilt through social support and a healthy lifestyle [17]. In particular, rejection and punishment should be avoided in childrearing to prevent vulnerability to suicidal behaviour [18,19,20,21]. Minding the psychological aspects of building and rebuilding resilience to suicidality might be a way forward in preventing suicide. There is a gap in the research regarding answering the question of how to improve assessments and interventions, and of integrating psychological theories and research in the formation of assessments and interventions to prevent suicide. This study is, to our knowledge, the first one to investigate the symptoms and all DSM diagnoses among children and young adults in a national cohort of those who died by suicide in one year.

Therefore, we wanted to learn more about the frequencies of symptoms and diagnoses noted in the medical records prior to suicide in a population of children and young adults and analyse the findings in relation to contemporary psychological theories and psychological research of suicidality regarding the psychology linked to suicidality, in order to formulate clinical implications about assessments and interventions to prevent suicide.

### Aim

The aim of this study was to gain a better understanding of the symptoms and diagnoses prior to suicide in children and young adults, as documented in their medical records, specifically (1) to measure the frequencies of symptoms and diagnoses prior to suicide in a group of children, in a group of young adults, and among all individuals aged under 25 years; (2) to measure the frequencies of symptoms and diagnoses in males and females within all the individuals under 25 years; and (3) to apply psychological theories about suicidality in children and young adults to the findings, in order to inform clinical implications.

## 2. Materials and Methods

This study is part of the nationwide project Retrospective Investigation of Health Care Utilization of Individuals who died by Suicide in Sweden 2015, Lund University, Sweden [22,23]. The data are stored at Forum South, Clinical Studies, in collaboration with Lund University. This medical record review covers all individuals who died by suicide in a single year. The current study focused on the symptoms and diagnoses prior to suicide in children and young adults.

### 2.1. Sample

The study cohort, which has been described previously in a study regarding suicidal communication [23], included 114 children (0–17 years) and young adults (18–24 years) who died by suicide in Sweden during 2015 [23], corresponding to all suicides in that age group during that year [24]. The gender distribution among the 114 individuals aged 24 and under were 71 males and 43 females (13 boys, 11 girls, 58 young men, and 32 young women) [23]. In this cohort, approximately 90 percent, or 102 of 114 individuals, had visited a health care setting within 24 months prior to their death; approximately 80 percent, or 93 of 114 individuals, had visited within a year; and approximately 60 percent, or 64 of 114 individuals, had visited within the last month prior to their suicide. Contact with psychiatric services was noted during the final two years of life in 60 percent of the individuals; approximately 70 percent had contact with primary care and 55 percent had contact with specialized somatic health care during this time period [23].

### 2.2. Data Collection Process

Data were collected from medical records covering the 24 months preceding each individual’s death by suicide. In Sweden, deaths by suicide are routinely subject to police investigation and, in the vast majority of cases, to medico-legal forensic examination; however, only cause-of-death data from the National Cause of Death Register were used in this study [24]. Data were available from all public records and efforts were made to obtain records from private clinics; few difficulties in obtaining non-governmental health care records were reported [22,23]. A structured investigation protocol was constructed, based on guidelines from the Swedish Psychiatric Organisation [25]. Each of the 21 Swedish counties appointed their record reviewers, 49 in total, who were trained to use the investigation protocol. The research group provided a manual with frequently asked questions and was available for further questions. The record review took place in 2016–2020. The data used in the present study were based on notes from the last visit with any health care professional, the last visit to a physician, and the last visit to in-patient care as noted by health care staff [23].

### 2.3. Variables

Demographic variables about gender and age were used. Gender was based on the Swedish identity number indicating male or female at the time of death. None in this cohort had a registered gender change [1].

Symptoms of both mental and physical health were included (depressive symptoms, anxiety symptoms, sleeping problems, symptoms of crisis reaction, symptoms of amnesia/disorientation, psychotic symptoms, other mental and behavioural symptoms, pain, loss of appetite, unintentional weight loss, gastrointestinal complaints, fatigue symptoms, neurological symptoms, dizziness, cardiovascular symptoms, hypertension), as were health issues, including sleep problems, breathing problems, urinary tract problems, symptoms of infection, undefined ongoing abuse, alcohol problems over the last three months, the misuse of hypnotics or sedatives over the last three months, the misuse of other drugs over the last three months, the influence of alcohol during the last suicide attempt, and visits to a drug clinic during the twelve months prior to suicide.

The diagnostic variables according to ICD-10 2015 included all the main chapters (I-XXII. Individuals could have more than one diagnosis recorded. The diagnoses were not prioritised or collapsed; each ICD-10 diagnostic category was coded and analysed as a separate binary variable (presence/absence). The comprehensive diagnostic approach was intentional and aligned with the study’s suicidality-focused aim to question the clinical utility of diagnosis-centred frameworks for suicide prevention in youth.

For chapter V, diagnoses of mental and behavioural disorders, all the subcategories were included on the first level: F0—organic, including symptomatic, mental disorders; F1—mental and behavioural disorders due to psychoactive substance use; F2—schizophrenia and schizotypal and delusional disorders; F3—mood (affective) disorders; F4—neurotic, stress-related, and somatoform disorders; F5—behavioural syndromes associated with physiological disturbances and physical factors; F6—disorders of adult personality and behaviour; F7—mental retardation (intellectual disabilities); F8—disorders of psychological development; and F9—behavioural and emotional disorders, with the onset usually occurring in childhood and adolescence, and unspecified mental disorders. Included on the second level of subcategories under F8 was F84—pervasive developmental disorder, including childhood autism and Asperger syndrome, and under F9 was F90—hyperkinetic disorders, including a disturbance of activity and attention. 

### 2.4. Data Analysis

Descriptive statistics were calculated for all variables (frequencies and proportions). Chi-squared tests were used for significance at the 95 percent level between males and females, and between the age groups of children and young adults. Significant differences in the frequency of the variables regarding gender and age were calculated. The study was primarily descriptive. Chi-squared tests were used only for exploratory comparisons between the age groups and genders when cell sizes permitted. In cases of small numbers, no statistical testing was performed, and the results were reported descriptively. IBM SPSS Statistics version 28.0.1.0 (142) was used for the analysis. In accordance with EU data protection regulations (GDPR), results involving very small cell counts (*n* ≤ 3) were not reported to reduce the risk of the indirect identification of individuals. No individual-level data were excluded from the analyses.

### 2.5. Ethical Considerations

An ethical review was not relevant in the Retrospective Investigation of Health Care Utilization of Individuals who died by Suicide in Sweden 2015 because it did not include living human participants according to the Swedish Act Concerning the Ethical Review Involving Humans (203:460) and to an advisory statement from the Regional Ethical Review Board of Lund, Sweden (ethical review decision No. 2017/234). The inclusion of the data from Stockholm County was approved by the Swedish Ethical Review Authority (ethical review decision No. 2019-02792).

## 3. Results

### 3.1. Symptoms Prior to Suicide

Table 1 shows the symptoms, including mental and physical health issues and alcohol and illegal substance use, recorded during the last visits of the individuals to psychiatric services, primary health care, and specialized somatic health care; the last visit to a health care professional, the last visit to a physician, and the last visit to in-patient care noted by health care staff 24 months prior to suicide are included. The most frequently noted symptoms were depressive symptoms, anxiety symptoms, and pain, which were each noted in about a fourth of the children and young adults. Sleeping problems and ongoing abuse were noted in approximately a fifth. Fatigue symptoms and other mental and behavioural symptoms were noted in approximately a sixth. Symptoms of infection, visits to a drug clinic within the last 12 months, breathing problems, and gastrointestinal problems were noted in about a tenth. The rest of the symptoms were noted in less than ten percent, such as neurological symptoms (not dizziness), a crisis reaction, loss of appetite, psychotic symptoms, urinary tract problems, amnesia/disorientation, dizziness, cardiovascular problems (not hypertension), alcohol problems in the last three months, other drug use in the last three months, and being under the influence of alcohol during the last suicide attempt. Unintentional weight loss, hypertension, and the misuse of hypnotics or sedatives in the last three months were noted in small numbers.

A fourth of the children had a record of fatigue, while a sixth had symptoms of an infection and depressive symptoms noted. The rest of the symptoms in the group of children were present in low numbers, meaning three children or fewer; those numbers are not specified in Table 1. This means that the numbers in the group of young adults must be hidden in the table, due to these symptoms exhibiting small numbers in the group of children. In the group of young adults, a third had a notation of depressive symptoms, approximately a tenth had fatigue, and the same proportion was noted for infection. Amnesia/disorientation, psychotic symptoms, cardiovascular symptoms, urinary tract problems, other drug use in the last three months, and being under the influence of other drugs were noted in seven percent or less. There were no significant differences between males and females regarding symptoms prior to suicide.

### 3.2. Diagnoses Prior to Suicide

Table 2 shows the diagnoses prior to suicide in children and young adults. Mental and behavioural diagnoses were noted in approximately 60 percent. The mental and behavioural disorders were divided into subcategories; see Table 3. The two chapters that registered symptoms not classified as diseases, disorders, or syndromes—symptoms, signs, and abnormal clinical and laboratory findings not elsewhere classified, and factors influencing health status and contact with health services—were noted in approximately a fourth of the children and young adults. Injury, poisoning, and certain other consequences of external causes and external causes of morbidity and mortality were also noted in a fourth. The rest of the chapters of diagnoses were observed in approximately ten percent or less. There were no significant differences between children and young adults regarding any of the diagnoses, and a few of them were not countable due to exhibiting small numbers in one of the groups. There were significant differences between males and females only in the chapter of diagnoses regarding mental and behavioural disorders: half of the males and approximately 80 percent of the females had a notation of at least one mental or behavioural disorder. 

#### Diagnoses of Mental and Behavioural Disorders Prior to Suicide

The mental and behavioural disorders are presented per subcategory; see Table 3. The results in the text are presented in the order of their magnitude. Neurotic, stress-related, and somatoform disorders and mood (affective) disorders were noted in approximately a third of the children’s and young adults’ medical records. Behavioural and emotional disorders with onset usually in childhood or early adulthood and unspecified mental disorders were noted in a fourth, and the subcategory of hyperkinetic disorders, including a disturbance of activity and attention, was noted in a fifth. Mental and behavioural disorders due to psychoactive substance use were noted in almost 20 percent. Behavioural syndromes associated with physiological disturbances and physical factors; disorders of psychological development; the subcategory of pervasive developmental disorders, including childhood autism and Asperger syndrome; and intellectual disability, registered as mental retardation, were noted in approximately ten percent each. Organic, including symptomatic, mental disorders were exhibited in small numbers along with schizophrenia and schizotypal and delusional disorders.

There were no significant differences between children and young adults. In the group of children, a fourth had a history of neurotic, stress-related, and somatoform disorders and a fifth had disorders of psychological development. The rest of the diagnoses noted in the group of children were zero or hidden in the table due to small numbers. In the group of young adults, a third had a notation of neurotic, stress-related, and somatoform disorders. Disorders of psychological development were noted in nine percent. Mental retardations were noted in four percent. The rest of the mental and behavioural disorders were hidden in the table due to these disorders exhibiting low numbers in the children or young adults.

There were no significant differences between males and females in the subcategories of mental and behavioural disorders, and the noted diagnoses demonstrated diversity and heterogeneity in the youth who died by suicide. Neurotic, stress-related, and somatoform disorders were noted in a fourth of males and in approximately 40 percent of females. Mood (affective) disorders were noted in a fourth of males and in approximately a third of females. Behavioural and emotional disorders with an onset usually in childhood or early adulthood and unspecified mental disorders were noted in approximately 20 percent of males and in approximately 30 percent of females, and the subcategory of hyperkinetic disorders, including a disturbance of activity and attention, were noted in a fifth of males and in a fourth of females. Behavioural syndromes associated with physiological disturbances and physical factors were noted in six percent of males and twenty-one percent of females. Mental and behavioural disorders due to psychoactive substance abuse were noted in almost a fifth of males and females. Disorders of adult personality and behaviours were noted in seven percent of males and in fourteen percent of females. Disorders of psychological development were noted in ten percent of males and in fourteen percent of females, and the subcategory of pervasive developmental disorders, mainly childhood autism and Asperger syndrome, was noted in approximately ten percent of males and females. Organic, including symptomatic, mental disorders had small or no numbers, along with schizophrenia and schizotypal and delusional disorders.

## 4. Discussion

Diagnoses were rare in all the chapters of ICD-10 except for mental and behavioural diagnoses. Mental and behavioural symptoms and diagnoses were more common. The most common symptoms were depressive symptoms (28%), anxiety symptoms (26%), and pain (25%). The children’s main symptoms were fatigue (25%), depressive symptoms (17%), and symptoms of infection (17%). Mental and behavioural diagnoses were noted in approximately 60 percent, with the main diagnoses being mood (affective) disorders (29%) and neurotic, stress-related, and somatoform disorders (32%). The symptoms and diagnoses did not sum up to a clear picture of common factors prior to suicidality; rather low levels of diagnoses or symptoms formed the general picture in this cohort of children and young adults. Werbart Törnblom et al. [26] studied individuals between 10 and 25 years of age that died by suicide and found that the pathway to death by suicide included a lack of steady relationships, sexual trauma, depression, autism, being a psychiatric patient, recent stressors in daily life, low levels of adaptive coping, and alcohol or drug use during suicide.

The most represented diagnoses of mental and behavioural disorders in the whole cohort were mood (affective) disorders and neurotic, stress-related, and somatoform disorders, with both of these categories being noted in approximately a fourth. Among the group of children, the most common diagnoses of mental and behavioural disorders were neurotic, stress-related, and somatoform disorders, which were noted in a fourth, and disorders of psychological development, which were noted in a fifth. The significant difference between males and females regarding mental and behavioural diagnoses mirrors the percentage of males and females that visit psychiatric services. Fifty-four percent of males and seventy percent of females in this study cohort visited psychiatric services during their last visits within 24 months prior to suicide [23]. An analysis of the most common symptoms and diagnoses noted in the medical records gives a picture of parts of the group having been sad, stressed, tired, or in pain.

The causes of suicidality can be formulated in terms other than the symptoms adding up to meet the diagnostic criteria of mental and behavioural disorders [26]. The result of this study underscores the need for a focus other than symptoms and diagnoses in identifying youth at risk of suicidality. Notably, anxiety symptoms were noted in the medical records of only one quarter of the suicide decedents in our study, which contrasts with the proportion (39%) reported in the mixed-age national cohort of suicides that occurred in Sweden in 2015 [27].

The findings of this study on the proportions of symptoms and diagnoses indicate that the clinical focus needs to be broadened from symptoms and diagnoses to diminish the number of suicides. The majority had mental and behavioural problems noted, which indicates that they did not feel mentally well. However, splitting it up into specific symptoms and diagnoses results in low numbers for each symptom and diagnosis. This indicates that symptoms and diagnoses as such do not identify suicidality, implying a need to start working in another way. A history of diagnoses and symptoms prior to suicide was neither a sufficient nor a necessary condition to prevent, predict, explain, or understand the phenomenon of suicidality. From a theoretical and clinical perspective, suicidality in children and young adults is widely understood as transdiagnostic and heterogeneous. Previous research has shown that suicide risk is not confined to any single psychiatric diagnosis and that a substantial proportion of individuals who die by suicide either lack a severe mental disorder or present with diverse and overlapping diagnostic profiles. By including all ICD-10 diagnostic categories, we were able to illustrate this diagnostic diversity and demonstrate that no single diagnostic pattern predominates prior to suicide in this population. The inclusion of all diagnoses allowed us to empirically support one of the key conclusions of the study: that symptoms and diagnoses, as documented in routine health care, are not sufficient to identify suicidality in children and young adults. The finding that mental and behavioural diagnoses were common, but highly heterogeneous and often non-specific, underscores the need to broaden the clinical assessment beyond diagnostic checklists toward psychological, social, and developmental factors relevant to suicidality. The implementation of a psychological perspective in the discursive understanding of suicidality parallel to, or as an alternative to, the psychiatric use of mapping symptoms and diagnoses is of interest, and it should be investigated to determine if it could be fruitful in the work of preventing suicide in youth.

### 4.1. Clinical Implications

What happens prior to the mental and behavioural symptoms, and prior to the internalized and externalized symptoms? What needs to be socially and psychologically rebuilt? These might be relevant questions to ask, if we are going to understand what the symptoms are symptoms of. Suicidality is not simply an epiphenomenon, a symptom or a consequence, of a psychiatric disorder [28]. Family problems and intimate relationship problems were the most common situations among suicide descendants; they met challenges to family and life stressors and acute crises, regardless of the presence of a mental health diagnosis [29]. It is important to intervene through building resilience to challenging life situations and emotions, to prevent or diminish suicidal thinking and behaviour. Perez et al. [12] underscored that attention should be given to stressful life events, such as interpersonal problems, legal issues, family conflict, and substance rehabilitation. Therapy can address the ongoing stressors as well as the accumulation of recent and historical stressful life events, and also improve the ability to cope with stressful life events and to manage negative emotions constructively, thereby preventing suicide [12]. Enhancing self-esteem and self-efficacy improves psychological well-being, which can, in turn, improve mental health [30].

Means restrictions as well as the identification of young adolescents before their first suicide attempt are important [30]. McKean et al. [31] found that 70 percent of their study individuals in the age group of 10–24 years died during their first attempt, attracting medical attention. Health care utilization is not enough; screening for suicide risk needs to expand to other settings, including schools and primary care, which can help identify youth at risk for suicide [32], as well as facilitate interventions to prevent suicide. Since it is difficult to identify youth specifically at risk for suicide, the focus on coping with challenging life situations, ongoing or previous or both, and managing emotions constructively might be a way forward to help more youth and to prevent suicide. Age-related disparities in the symptomatology of suicidality need to be considered [17]. Marked impairment in multiple domains is associated with suicide-related outcomes; the youth suicidal population is heterogeneous [33].

It is problematic to view symptoms as diseases in psychiatry, since it might lead to overinterpretation [33], and more importantly, it might result in other important areas of problem-solving being overlooked, such as social problems and psychological problems. It might also result in people interpreting their problems as various kinds of psychopathology, thereby contributing to the increasing number of diagnoses [34]. Foulkes and Andrews [34] described this vicious circle as the prevalence inflation. Interpreting difficulties as mental health problems can lead to changes in self-concept, which in turn exacerbate symptoms and distress; improved recognition feeds overinterpretation [34]. Psychiatric diagnoses not only impact changes in the self-concept of patients, but they also influence the organization of psychiatric services. Brinkmann [35] discussed the problems of diagnostic psychiatry and pointed out that psychiatric manuals have a central role in pressuring modern health care towards standardization and manualization, downplaying professional judgment and favouring so-called evidenced-based knowledge that is often based on checklists.

To improve in reducing suicide rates, a reasonable way would be to aim for a multidimensional, holistic perspective, including social sciences and natural sciences, embracing the diversity among suicidal patients and finding solutions to their individual problems. Morgan [36] argued that the descriptive phenomenology that psychopathology has developed into needs to be allied with a deeper phenomenology. This includes attention to the meaning that people give to their experiences and an ability to endure when the meaning does not arise directly [35]. To avoid oversimplistic claims, Stein and Nesse [37] emphasized that it is important to hold the diagnoses lightly and embrace explanatory pluralism. These authors [37] were critical of the view that, for example, anxiety and depression are specific defects in a machine, arguing that this obscures the organic complexity of a living system [37]. An understanding of mental health as a relation between the inner and outer worlds of a person that synthesises biology and psychology [35] might lead us to a better understanding of youth suicidality that can be used to prevent suicide.

### 4.2. Limitations

The sample used in this study covered a national cohort of up to 25 years, with a particular year in Sweden giving generalisability, though our study did not have a control group. Medical record data have limitations regarding the risk of incomplete or inaccurate documentation, for example, due to wrong or incomplete diagnostics or mistakes during documentation. Due to the retrospective data collection process, the data could not be completed. Our findings cannot be extrapolated to settings with different types of access to health care for youth.

### 4.3. Research Implications

The implications for future studies include a recommendation to further explore the expression or lack of expression of children and young adults prior to suicide. Future studies will continue to incorporate various theoretical perspectives to further explore the plurality of youth suicidality.

## 5. Conclusions

Somatic symptoms and diagnoses were noted in very few of the children and young adults in their last visits to health care services within 24 months prior to suicide. Mental and behavioural symptoms and diagnoses were more common. Diagnoses and symptoms in medical records are not sufficient to uncover suicidality in children and young adults. We need to shift from a focus on symptoms and diagnoses to what happens prior to the symptoms and diagnoses, and to focus on the interventions needed to overcome those obstacles and reach longevity. A multidimensional holistic perspective is needed, with a focus both within and out of the clinic, aiming at understanding the individual causes of suicidality to find solutions to an individual’s problems and build resilience.

## Figures and Tables

**Table 1:** Symptoms, including mental and physical health issues and alcohol and illegal substance misuse, prior to suicide in children and young adults (N = 114). Number and percentage (%) of children (0–17 years, *n* = 24), young adults (18–24 years, *n* = 90), all males (*n* = 71), and all females (*n* = 43), with a record of symptoms at their last visits within 24 months prior to suicide.

Symptoms Prior to Suicide	Total *n* (%)	Children *n* (%)	Young Adults *n* (%)	Males *n* (%)	Females *n* (%)
Depressive symptoms	32 (28%)	4 (17%)	28 (31%)	19 (27%)	13 (30%)
Anxiety symptoms	30 (26%)	- ^1^	-	18 (25%)	12 (28%)
Sleeping problems	25 (22%)	- ^1^	-	14 (20%)	11 (26%)
Symptoms of crisis reaction	8 (7%)	- ^1^	-	-	- ^1^
Symptoms of amnesia/disorientation	4 (4%)	0 (0%)	4 (4%)	- ^1^	- ^1^
Psychotic symptoms	6 (5%)	0 (0%)	6 (7%)	4 (6%)	2 (5%)
Other mental or behavioural symptoms	18 (16%)	- ^1^	-	12 (17%)	6 (14%)
Pain	29 (25%)	- ^1^	-	18 (25%)	11 (26%)
Loss of appetite	8 (7%)	- ^1^	-	- ^1^	-
Unintentional weight loss	^_ 1^	- ^1^	- ^1^	0 (0%)	- ^1^
Gastrointestinal complaints	11 (10%)	- ^1^	-	4 (6%)	7 (16%)
Fatigue symptoms	18 (16%)	6 (25%)	12 (13%)	11 (15%)	7 (16%)
Neurological symptoms (not dizziness)	9 (8%)	- ^1^	-	-	- ^1^
Dizziness	5 (4%)	- ^1^	-	-	- ^1^
Cardiovascular symptoms (not hypertension)	5 (4%)	0 (0%)	5 (6%)	-	- ^1^
Hypertension	^_ 1^	0 (0%)	- ^1^	- ^1^	0 (0%)
Breathing problems	13 (11%)	- ^1^	-	8 (11%)	5 (12%)
Urinary tract problems	6 (5%)	0 (0%)	6 (7%)	- ^1^	- ^1^
Symptoms of infection	15 (13%)	4 (17%)	11 (12%)	8 (11%)	7 (16%)
Ongoing abuse, undefined	20 (18%)	- ^1^	-	13 (18%)	7 (16%)
Alcohol problems, last three months	5 (4%)	- ^1^	-	- ^1^	- ^1^
Misuse of hypnotics or sedatives, last three months	^_ 1^	0 (0%)	- ^1^	- ^1^	- ^1^
Misuse of other drugs, last three months	4 (4%)	0 (0%)	4 (4%)	- ^1^	- ^1^
Influence of alcohol during the last suicide attempt	5 (4%)	0 (0%)	5 (6%)	0 (0)	5 (12%)
Visit to drug clinic, last 12 months	14 (12%)	- ^1^	-	6 (9%)	8 (19%)

Differences between males and females were evaluated with chi-squared tests, and significant differences were not found in those cases where it was calculable. ^1^ Small numbers were omitted, in accordance with GDPR (EU, 2016/679), and chi-squared tests were not conducted due to small numbers in these cases.

**Table 2:** Diagnoses prior to suicide in children and young adults (N = 114). Number and percentage (%) of children (0–17 years, *n* = 24), young adults (18–24 years, *n* = 90), all males (*n* = 71), and all females (*n* = 43) with a record of diagnosis at their last visits within 24 months prior to suicide.

Diagnoses Prior to Suicide, ICD-10 2015	Total *n* (%)	Children *n* (%)	Young Adults *n* (%)	Males *n* (%)	Females *n* (%)
I—Certain infectious and parasitic diseases	7 (6%)	- ^1^	-	-	- ^1^
II—Neoplasms	- ^1^	- ^1^	- ^1^	- ^1^	- ^1^
III—Diseases of the blood and the blood-forming organs and certain disorders involving the immune mechanism	0 (0%)	0 (0%)	0 (0%)	0 (0%)	0 (0%)
IV—Endocrine, nutritional, and metabolic diseases	5 (4%)	0 (0%)	5 (6%)	- ^1^	-
V—Mental and behavioural disorders	70 (61%)	15 (63%)	55 (61%)	37 (52%) *	33 (77%) *
VI—Diseases of the nervous system	6 (5%)	0 (0%)	6 (7%)	- ^1^	- ^1^
VII—Diseases of the eye and adnexa	7 (6%)	- ^1^	-	- ^1^	-
VIII—Diseases of the ear and the mastoid process	- ^1^	0 (0%)	- ^1^	- ^1^	- ^1^
IX—Diseases of the circulatory system	- ^1^	0 (0%)	- ^1^	- ^1^	0 (0%)
X—Diseases of the respiratory system	12 (11%)	- ^1^	-	- ^1^	-
XI—Diseases of the digestive system	6 (5%)	0 (0)	6 (7%)	- ^1^	- ^1^
XII—Diseases of the skin and subcutaneous tissue	11 (10%)	- ^1^	-	4 (6%)	7 (16%)
XIII—Diseases of the musculoskeletal system and connective tissue	4 (4%)	- ^1^	- ^1^	- ^1^	- ^1^
XIV—Diseases of the genitourinary system	9 (8%)	- ^1^	-	- ^1^	-
XV—Pregnancy, childbirth, and puerperium	- ^1^	0 (0%)	- ^1^	0 (0%)	- ^1^
XVI—Certain conditions originating in the perinatal period	0 (0%)	0 (0%)	0 (0%)	0 (0%)	0 (0%)
XVII—Congenital malformations, deformations, and chromosomal abnormalities	0 (0%)	0 (0%)	0 (0%)	0 (0%)	0 (0%)
XVIII—Symptoms, signs, and abnormal clinical and laboratory findings not elsewhere classified	29 (25%)	5 (21%)	24 (27%)	17 (24%)	12 (28%)
XIX—Injury, poisoning, and certain other consequences of external causes	29 (25%)	- ^1^	-	19 (27%)	10 (23%)
XX—External causes of morbidity and mortality	24 (21%)	- ^1^	-	17 (24%)	7 (16%)
XXI—Factors influencing health status and contact with health services	26 (23%)	5 (21%)	21 (23%)	14 (20%)	12 (28%)
XXII—Codes for special purposes	0 (0%)	0 (0%)	0 (0%)	0 (0%)	0 (0%)

Differences between males and females were evaluated with chi-squared tests; * denotes significance at the 5% level. ^1^ Small numbers were omitted, in accordance with GDPR (EU, 2016/679), and chi-squared tests were not conducted due to small numbers in these cases.

**Table 3:** Diagnoses of mental and behavioural disorders prior to suicide in children and young adults (N = 114). Number and percentage (%) of children (0–17 years, *n* = 24), young adults (18–24 years, *n* = 90), all males (*n* = 71), and all females (*n* = 43) with a record of diagnosis at their last visits within 24 months prior to suicide.

Mental and Behavioural Diagnoses Prior to Suicide, ICD-10 2015	Total *n* (%)	Children *n* (%)	Young Adults *n* (%)	Males *n* (%)	Females *n* (%)
V—Mental and behavioural disorders in total	70 (61%)	15 (63%)	55 (61%)	37 (52%) *	33 (77%) *
F0—Organic, including symptomatic, mental disorders	- ^1^	0 (0%)	- ^1^	- ^1^	0 (0%)
F1—Mental and behavioural disorders due to psychoactive substance use	19 (17%)	- ^1^	-	11 (16%)	8 (19%)
F2—Schizophrenia and schizotypal and delusional disorders	- ^1^	0 (0%)	- ^1^	- ^1^	- ^1^
F3—Mood (affective) disorders	33 (29%)	- ^1^	-	18 (25%)	15 (35%)
F4—Neurotic, stress-related, and somatoform disorders	36 (32%)	6 (25,0)	30 (33%)	18 (25%)	18 (42%)
F5—Behavioural syndromes associated with physiological disturbances and physical factors	13 (11%)	- ^1^	-	4 (6%)	9 (21%)
F6—Disorders of adult personality and behaviour	11 (10%)	- ^1^	-	5 (7%)	6 (14%)
F7—Mental retardation (intellectual disability)	4 (4%)	0 (0%)	4 (4%)	- ^1^	- ^1^
F8—Disorders of psychological development	13 (11%)	5 (21%)	8 (9%)	7 (10%)	6 (14%)
F84—Pervasive developmental disorder, mainly childhood autism and Asperger syndrome	11 (10%)	- ^1^	-	6 (9%)	5 (12%)
F9—Behavioural and emotional disorders with onset usually occurring in childhood and adolescence and unspecified mental disorders	27 (24%)	- ^1^	-	15 (21%)	12 (28%)
F90—Hyperkinetic disorders, mainly a disturbance of activity and attention	24 (21%)	- ^1^	-	14 (20%)	10 (23%)

Differences between males and females were evaluated with chi-squared tests; * denotes significance at the 5% level. ^1^ Small numbers were omitted, in accordance with GDPR (EU, 2016/679).

## Data Availability

The data will be made available upon reasonable request.
